# Spatial abundance models and seasonal distribution for guanaco (*Lama guanicoe*) in central Tierra del Fuego, Argentina

**DOI:** 10.1371/journal.pone.0197814

**Published:** 2018-05-21

**Authors:** Celina E. Flores, Guillermo Deferrari, Leonardo Collado, Julio Escobar, Adrián Schiavini

**Affiliations:** 1 Centro Austral de Investigaciones Científicas (CADIC-CONICET), Ushuaia, Tierra del Fuego, Argentina; 2 Dirección General de Bosques, Secretaría de Ambiente, Desarrollo Sostenible y Cambio Climático, Ushuaia, Tierra del Fuego, Argentina; Universita degli Studi di Napoli Federico II, ITALY

## Abstract

Spatially explicit modelling allows to estimate population abundance and predict species’ distribution in relation to environmental factors. Abiotic factors are the main determinants of a herbivore´s response to environmental heterogeneity on large spatiotemporal scales. We assessed the influence of elevation, geographic location and distance to the coast on the seasonal abundance and distribution of guanaco (*Lama guanicoe*) in central Tierra del Fuego, by means of spatially explicit modelling. The estimated abundance was 23,690 individuals for the non-breeding season and 33,928 individuals for the breeding season. The factors influencing distribution and abundance revealed to be the elevation for the non-breeding season, and the distance to the coast and geographic location for the breeding season. The southwest of the study area presented seasonal abundance variation and the southeast and northeast presented high abundance during both seasons. The elevation would be the driving factor of guanaco distribution, as individuals move to lower areas during the non-breeding season and ascend to high areas during the breeding season. Our results confirm that part of the guanaco population performs seasonal migratory movements and that the main valleys present important wintering habitats for guanacos as well as up-hill zones during summer. This type of study would help to avoid problems of scale mismatch and achieve better results in management actions and is an example of how to assess important seasonal habitats from evaluations of abundance and distribution patterns.

## Introduction

The understanding of habitat requirements and population abundance is part of the knowledge necessary to tackle biological conservation targets [1, 2]. This information is generally considered in the design of protected areas [1], monitoring of population trends [3] and development of management practices [1, 2, 4]. For species living in seasonal environments and moving according to the availability of resources, recognizing the habitat combination used to accomplish their biological cycle is particularly important [1, 5]. In these cases, the challenge is to encompass sufficiently large spatial extensions to capture the range of resources variability on which the species may depend [6].

Theoretical developments of spatial ecology alongside improvement of statistical methodologies and computational software, as well as the increasing availability of spatially referenced environmental information, have allowed stressing the importance of the spatial context for the analysis of populations, especially in large-scale studies [7]. Spatially explicit modelling has become widely used to assess species-habitat relationships [8–11]. These modelling strategies have been mainly applied to predict species distributions and to know how they are affected by environmental factors [12, 13]. One of them is Density Surface Modelling, which builds spatial models of the relationship between abundance and environmental factors to estimate overall abundance [14, 15].

Both biotic and abiotic factors regulate the geographic distribution of species [16, 17]. But, the degree of influence by each kind of factor is scale dependent [6, 18, 19]. On large spatiotemporal scales, the abiotic factors generally act as the main determinants of a herbivore´s response to environmental heterogeneity [18, 19]. Therefore, several studies described that distributional patterns of ungulates are well explained by gradients of altitude [20, 21], soil fertility [22, 23], water availability [17, 24], rainfall [25–27] and by a combination of the two last [28].

Seasonal displacements in response to elevation have been reported for wild ungulate species that inhabit mountain zones [29–33]. The effect of continentality in habitat selection was also reported for the caribou [34]. Seasonal movements were reported for the guanaco (*Lama guanicoe*), a large South American herbivore [35], in environments with pronounced elevation gradients like the Chilean sector of Tierra del Fuego [36, 37], but are unknown for the Argentinean sector of Tierra del Fuego, an area with altitudinal ranges from 0 to 1,400 masl [38, 39]. The usual explanation for these movements refers to the severity of climatic conditions on higher zones during winter [37, 40], which can also be accentuated in inland zones far from the moderating effect of the sea [41]. Knowing the extent to which environmental factors drive the guanaco distribution pattern would help to understand their seasonal habitat requirements.

Guanaco populations have declined in South America since the 1,800 s, from numbers reaching about 30 million originally to the current estimates of about 600 thousand animals [42]. Currently, the guanaco is a specie included in Appendix II of CITES and managed by a National Management Plan [43]. The last abundance figures from Argentinian Tierra del Fuego were produced more than 20 years ago [44]. Then, the aim of this paper is to update this information and to identify seasonal patterns of abundance distribution and habitat requirements, a type of information essential to drive conservation management actions [1]. This study may also be useful to recognize habitats of seasonal importance in other species of large herbivores that respond to environmental heterogeneity with migratory displacement [5].

In this study we built spatially explicit models of guanaco abundance by the approach of Density Surface Modelling, relating count data with abiotic environmental factors [14]. The objectives of this study were: 1) to estimate the abundance of guanaco in the center of the Isla Grande de Tierra del Fuego and, 2) to analyze their seasonal distribution in relation to elevation, distance to the coast (as a proxy of continentality) and geographic location to provide information about seasonal habitat requirements and movements.

## Materials and methods

### Ethics statement

This study was conducted with wild free-ranging animals and was completely observational. Observations were conducted from a helicopter and therefore no handling of animals took place. Flight was performed over private lands and over a provincial protected area lands. As the provincial government was the beneficiary of the grant, permission was granted for flying over protected areas. No special permit was needed for flying over private lands.

### Study area

**Fig 1:**
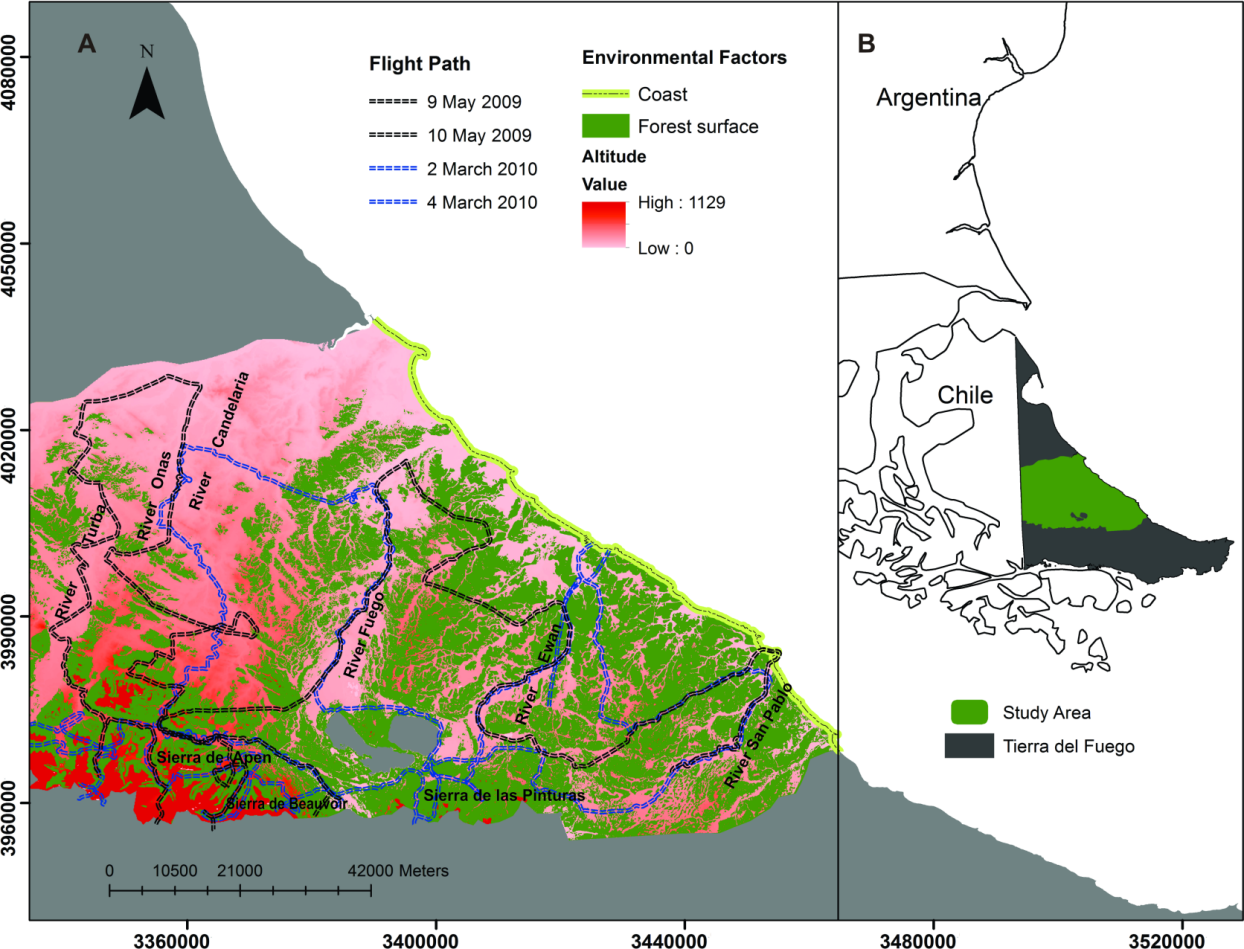
Study area and flight paths. (A) Environmental factors of the study area, places of common references (the valleys of most important rivers and hill zones) and paths. (B) Location of study area in the province of Tierra del Fuego, Argentina.

The study area covers the centre of the Argentinian portion of the Isla Grande de Tierra del Fuego (53° 48' - 54° 32 ' S and 66° 34' - 68° 36' W) with an area of 7,107 km^2^. The called Ecotone Ecological Region predominates in the area, representing a transition between forested ranges and the grasslands of the Magellanic Steppe [45, 46] (Fig 1). The climate is temperate cold, with an average annual rainfall of 400–500 mm evenly distributed throughout the year [45]. The average temperature of the coldest month is -4°C and of the warmest month is 10°C [47]. Rainfall increases to the South while the temperature decreases, with persistent snowfall in the southern areas during winter [45, 46]. The forest vegetation is composed by tree species of the genus *Nothofagus* sp. (Antarctic beech, *Nothofagus antarctica*, Southern deciduous beech, *Nothofagus pumilio* and Southern evergreen beech, *Nothofagus betuloides*) and occurs in patches decreasing in size towards the north, from the range to the steppe, giving rise to pastures between the forest mass. The physiognomy of grasses varies according to the terrain. In high and less drained areas the *Festuca gracillima* dominate, while in depressed areas near the water table, grasses of the genus *Carex sp*. (*Carex magellánica*, *Carex microglochin* and *Carex macloviana*) predominate. Other important grasses are *Poa pratensis*, *Phelum sp*., *Agropyrum magellanicum*, *Hordeum sp*., *Trisetum sp*., *Osmorhiza chilensis* and *Galium aparine*. In degraded areas, *Empetrum rubrum* and *Bolax gummifera* predominate [45]. The landscape is shaped to the centre and to the north by hills of 170–130 masl, and by wide valleys with general SW-NE orientation. The hills change course in a fan form, from W-E to NW-SE. Further to the south of the study area, a range system develops with altitudes between 1,000 and 600 masl [39].

### Field work

Sampling was carried out during the breeding season (March 2 and 4, 2010) and during the non-breeding season (May 9 and 10, 2009), to account for two contrasting moments both in weather and for the biology of the species, given its social reproductive behaviour. According to Ortega and Franklin [40], the reproductive period ranges from mid-October to late March, when populations are structured hierarchically and calving and copulation occur. Between April and early October, the social structure is relaxed and the guanaco is distributed mainly in large mixed groups and groups of females with calves.

Guanaco groups were recorded from aerial surveys following a strip-transect methodology [48], carried out on board a Robinson R44 helicopter flying 100 m above ground level and at a constant speed of 60 knots. The helicopter allowed wide visibility of animals by its slow speed of 60 knots and low flight altitude [49]. During the flight, the main observer located at the front left seat of the helicopter photographed guanaco groups with a camera (12Mp, Canon Rebel XT). The pilot and a third observer helped the main observer detect guanaco groups. Photographs were georeferenced combining the time of capture and a GPS record obtained using a GPS device (I-GotU GT-120) with the software Trip PC.

A bandwidth of 1400 m (700 m at each side of the helicopter) was defined as the limit for our strip transect and all the pictures taken in subsequent surveys were assigned to this bandwidth, where the detection probability was assumed constant and equal to 1 [50]. The 700 m bandwidth was defined analyzing a set of pictures with the procedure described in S1 File. The helicopter surveys covered the elevational gradient and the variability represented by the distance to the coast and geographic location (Fig 1), a requirement to reduce spurious model results in Density Surface Modelling.

The undulating features of the relief and the presence of patches of forest may represent an obstacle to visualize the guanaco. However, guanacos perform daily movements between forest areas and pastures. During the night they seek shelter in the forest cover, while during the day they leave to the open areas of pastures to feed and remain vigilant [36]. Taking advantage of this behaviour, the surveys were conducted between 11 am and 15 pm mostly in open areas, assuming that all the animals were out of the forest and visible at that time.

### Modelling

To assess guanaco distribution abundance we modelled the response of guanaco abundance to environmental factors by spatially explicit modelling. The best model was then used to estimate the guanaco abundance when applied to a prediction grid of covariates.

For the modelling, a series of segments extracted from the flight path was used as the sampling unit. The segments were obtained by dividing the strip-transect every 1400 m in length, equivalent to the bandwidth, achieving an approximately square surface of 1,96 km^2^ [14]. In this way, each segment summarizes the guanaco counts and the environmental factors evaluated over it. The segment edition and assessment of the environmental factors were performed with the software ArcGIS 10.3.1 (ESRI). The elevation factor was summarized as the average elevation value for the surface covered by the segment, obtained from a product of SRTM-Non Void Filled Digital Elevation Model of 90 m resolution (http://Earthexplorer.usgs.gov). The distance to the coast was calculated from the central position of each segment, based on coastal boundary information provided by the *Secretaria de Ambiente*, *Desarrollo Sustentable y Cambio Climático* of the province of Tierra del Fuego. The percentage of area covered by forest at each segment was obtained from a forest cover mask also provided by the provincial government. As guanacos can be detected in small clearings and very close to the forest from the air, segments even with a forest cover less than 95% were included in the analysis.

The effect of environmental factors on guanaco abundance was assessed by modelling for the breeding season and for the non-breeding season separately. To avoid multicollinearity, the correlation between the Average Elevation and Distance to the Coast were evaluated at each time point with the Pearson correlation coefficient. It was considered that a value of | r |> = 0.4 would be high enough to analyze the effect of both factors on separate models [51, 52].

For the modelling, we used GAM (Generalized Additives Models) with a Tweedie overdispersion distribution and logarithmic link function. The degrees of freedom were adjusted for each model according to the Maximum Restricted Likelihood (REML) method, choosing between 3 and 19 knots (k = 4–20) according to the lowest REML value. The best model for each period was chosen as the one with the lowest REML value. For the selected models, spatial autocorrelation was evaluated and corrected using spatial autocorrelation structure with GAMM (Generalized Mixed Additives Models) [14], in cases where the correlation percentage was greater than 15%. In those cases, we evaluated the explanatory capacity of the models comparing the variance between GAM model and its later GAMM model with corrected autocorrelation [14]. Those values showed us the fit for GAMM respect to GAM obtained before, whose explained deviance is known. The modelling procedure was implemented with the *dsm* package in an R environment (http://github.com/DistanceDevelopment/dsm) [14]. In the most recent versions, the *dsm* package allows estimating the detection function using a Uniform detection function, producing a strip transect estimation, as was done in Dellabianca *et al*. [50].

### Estimation

To estimate abundance for each period in the study area, we built a prediction grid with 1,96 km^2^ cells to which we applied each selected model. Each cell contained the value of the environmental factors considered: average elevation, distance to the coast and geographical location (as Longitude and Latitude in projected coordinates x and y), all expressed in meters. Cells with a forest cover area equal to or greater than 95% were excluded for the estimation, with the same criterion as for the segment exclusion. Applying the best models over the prediction grid we obtained an abundance estimation for each cell as well as the value of its coefficient of variation [14]. This allowed evaluating the spatial distribution of both the abundance and its variation. The total abundance of the study area was obtained adding up the abundance of all the cells and the average-values of density of individuals/km^2^ were calculated by dividing by the surface of the segment.

## Results

Four aerial surveys were carried out covering a total of 1,232 km of survey flight path. A total of 6,282 guanacos were recorded from 3,783 georeferenced photographs (3,190 individuals in the non-breeding season and 3,092 individuals in the breeding season) (Table 1). The average elevation and distance to coast presented significant correlation in both seasons (Pearson correlation coefficient: |r| = 0.69 in the non-breeding season, p<0.001 and |r| = 0.61 in the breeding season, p<0.001). For this reason, these covariates were evaluated in separate models. According to the criterion of segment exclusion from the area covered by forest, 8 segments for the non-breeding season and 6 for the breeding season, as well as 211 cells of the prediction grid were excluded from the analysis. As a result, the prediction grid presented 3,415 cells by the analysis.

**Table 1:** Sample effort, photographic records and number of guanacos registered by aerial survey.

Season	Aerial Survey	Effort (km)	Photographic Records	Registered Guanacos
**Non-Breeding**	10-May-09	294	842	1,618
	09-May-09	289.24	969	1,572
**Breeding**	02-Mar-10	295	923	1,406
	04-Mar-10	353	1,049	1,686

km. Distance traveled in kilometers.

The best models included average elevation and geographic location in non-breeding season and distance to the coast and geographic location in the breeding season (Table 2). Both models presented the lowest value of REML and highest explained deviance (Table 2). However, the best model of the non-breeding season presented high spatial autocorrelation of about 25% until 1,400 m of distance and 15% until 2,800 m of distance. This autocorrelation was corrected applying a correlation structure of order one until the second segment with GAMM. In this new model, geographic location resulted not significant and was eliminated. Thereby, average elevation remained as the only environmental factor for best model in the non-breeding season.

**Table 2:** Statistical parameters of models proposed and model selected (GAMs) by season.

Model	Explined deviance (%)	REML	Knots
**Non-Breeding Season**			
AA*** + GL***	**26.2**	**1,091.30**	**5–7**
DC + GL***	21.1	1,099.70	3–18
GL***	21.1	1,101.80	18
DC:GL***	24.1	1,102.10	12
AA:GL***	16.2	1,103.10	5
AA***	8.87	1,109.90	4
DC***	5.85	1,125.70	17
**Breeding Season**			
DC** + GL***	**24.2**	**1,232.30**	**4–9**
AA* + GL***	23.3	1,233.90	4–18
GL***	21.5	1,235.80	19
AA:GL***	12.5	1,242.90	6
DC:GL***	12.1	1,245.90	5
AA***	10	1247.4	13
DC***	7.25	1253.3	10

The best model selected by season is in bold.

References: AA, average altitude; GL, geographic location; DC, distance to coast.

Significance codes: <0.001

‘***’ 0.001

‘**’ 0.01

‘*’ 0.05 ‘.’ 0.1 ‘ ‘ 1.

**Fig 2:**
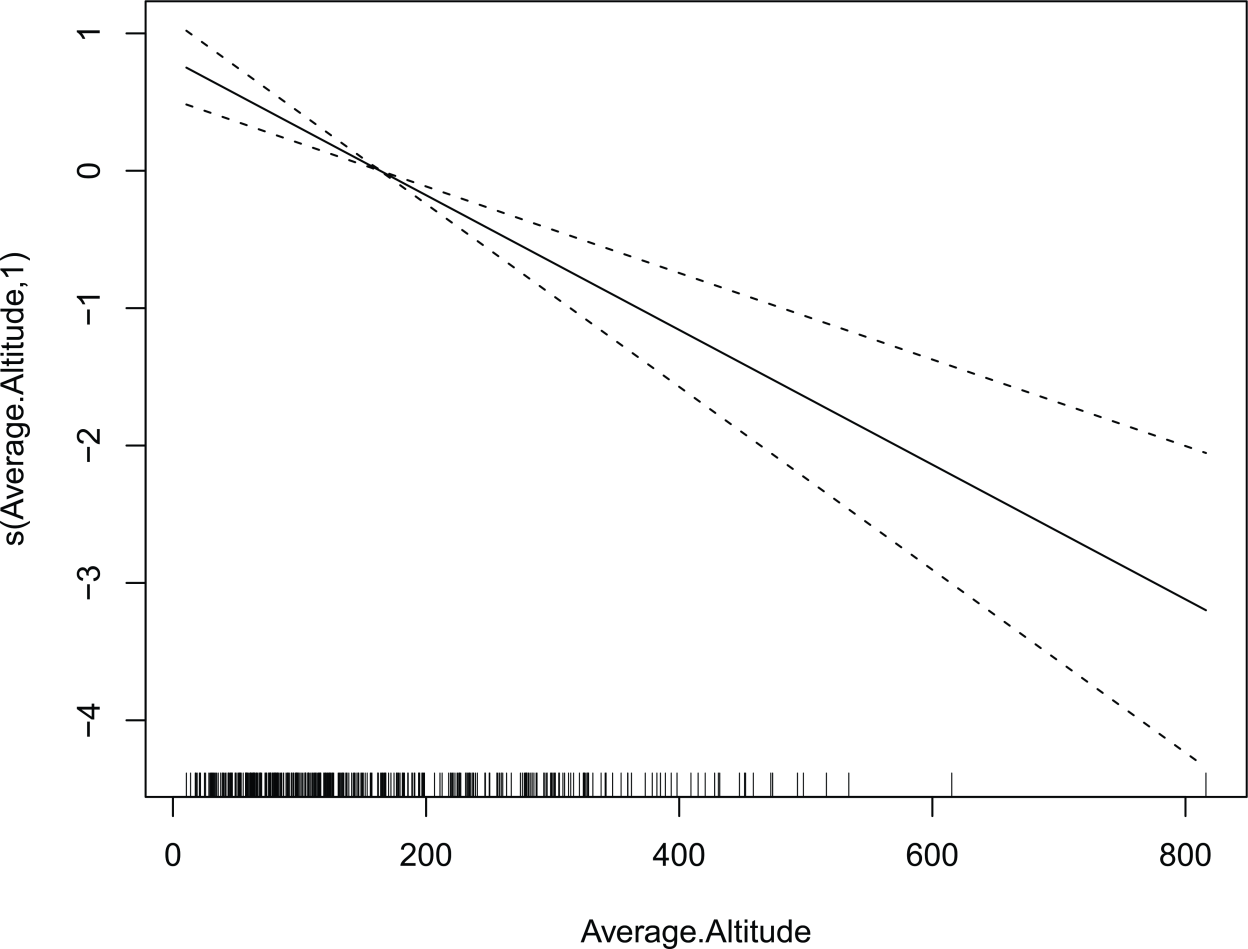
The estimated smooth term of the best model for guanaco abundance in the non-breeding season. The solid line represents the smoother for average elevation (expressed in meters) and the dotted lines are 95% point-wise confidence bands. The number in brackets in “s” is the estimated degree of freedom of the smooth term.

**Fig 3:**
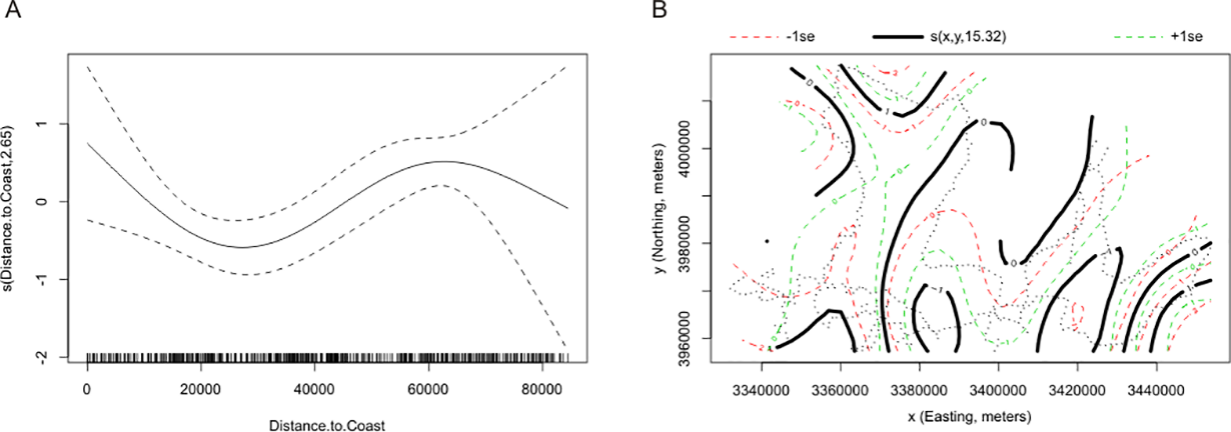
The estimated smooth term of the best model for guanaco abundance in the breeding season. (A) The solid line represents the smoother for Distance to Coast (expressed in meters) and the dotted lines are 95% point-wise confidence bands. The number in brackets in “s” is the estimated degree of freedom of the smooth term. (B) The black solid line represents the smoother for Geographic Location (y = Northing, x = Easting, expressed in meters). The green and red dotted lines are 95% point-wise confidence bands. The number in brackets in “s” is the estimated degree of freedom of the smooth term.

According to the best models, the guanaco abundance decreased with the increasing average elevation in the non-breeding season (Fig 2). In the breeding season, guanaco abundance increased toward zones far and nearby to coast (Fig 3A) and increased towards the north, east and southeast of the study area in relation to geographic location (Fig 3B).

The estimated abundance resulted in 23,690 individuals (CV = 15%) in the non-breeding season and 33,928 individuals (CV = 14%) in the breeding season. In turn, the average density value obtained was 3.54 ind/km^2^ for the non-breeding season and 5.07 ind/km^2^ for the breeding season. The coefficient of variation by GAM selected in the non-breeding season was 12%, a lower value respect to GAMM with corrected autocorrelation (15%), showing a similar fit and explicative capacity.

**Fig 4:**
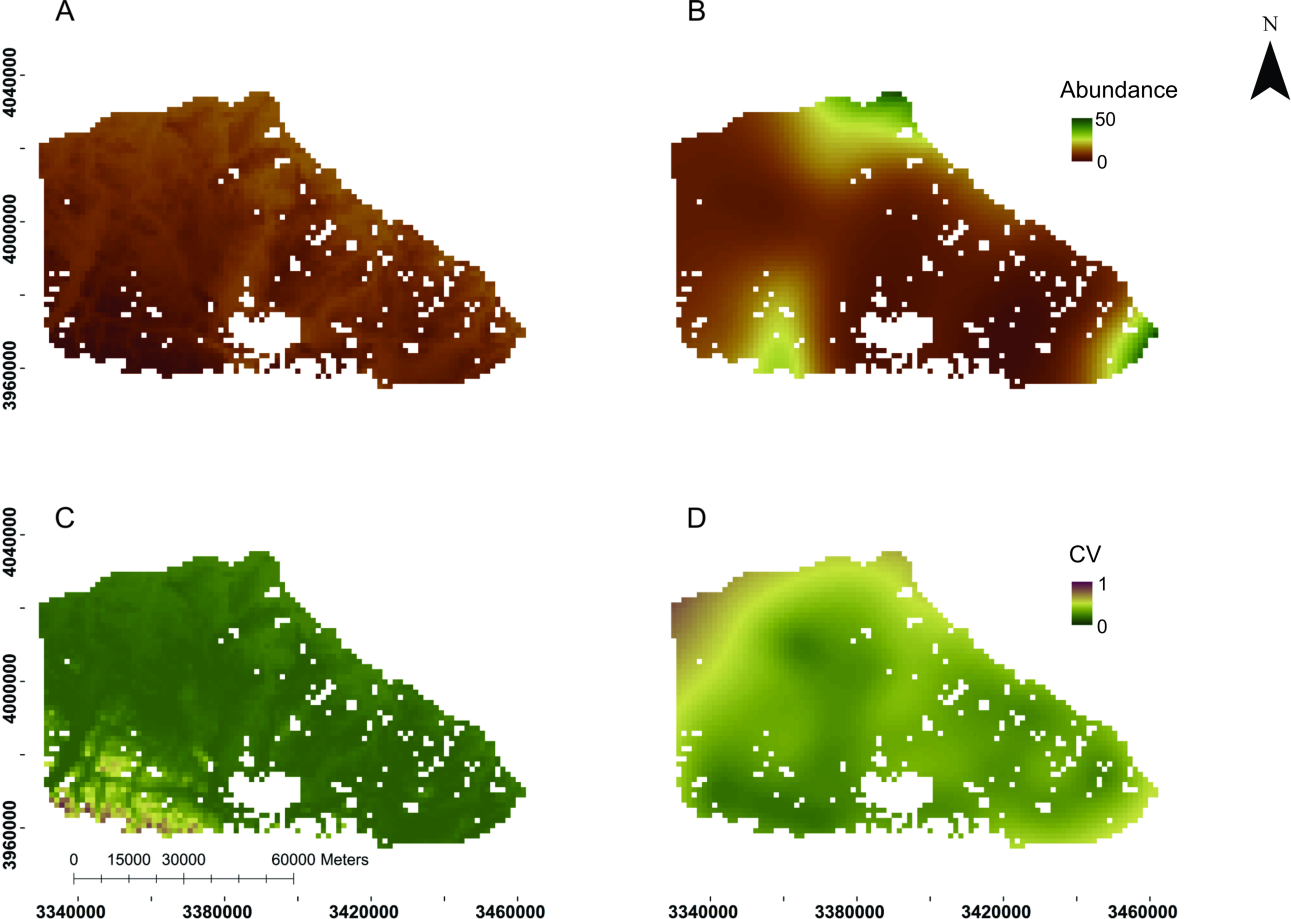
Map of seasonal abundance distribution and uncertainty for guanaco. Abundance distribution maps for the (A) non-breeding season and (B) breeding season. Distribution of coefficient of variation (CV) by the selected model in the (C) non-breeding season and in the (D) breeding season. In all panels, each cell corresponds to 1,96 km^2^ according to obtained in prediction grid.

The abundance was more heterogeneously distributed in the non-breeding season (Fig 4A) than in breeding season (Fig 4B). But, the Northeast and Southeast zones revealed high densities in both seasons. In turn, the Southwest zone specially presented an opposite seasonal pattern, with low densities in the non-breeding season and high densities in the breeding season. The coefficient of variation per-cell indicated an increase of model uncertainty towards the Southeast in the non-breeding season (Fig 4C) and toward North, Northeast and Southeast in the breeding season (Fig 4D).

## Discussion

The guanaco densities we report are of the same order of magnitude as previous estimates for the Argentinian portion of the Isla Grande de Tierra del Fuego. Bonino and Fernandez [53] reported in 1988 a maximum density of 2.8 ind/km^2^ for August and a minimum density of 0.9 ind/km^2^ for November by means of terrestrial surveys made by roads. Montes [44] reported for 1995, an average density of 1.73 ind/km^2^ for November, by means of aerial surveys. However, comparison of these estimates with ours is not possible, because of differences in methodologies, and because previous works are deficient in experimental design and lack measurement error.

To compare our results with those reported for other areas of the guanaco distribution range, we considered studies with reported measures of error of up to 30%. Our densities are in accordance with reports for Argentinian Patagonia (ranging between 0.95–7.56 ind/km^2^) [54–56] and for the Provincial Park Ischigualasto (0.12 ind/km^2^) [57], although other studies reported larger figures (ranging 3.25–26 ind/km^2^) respectively, for other protected areas [37, 58, 59]. Of special interest is the study on the Chilean sector of Tierra del Fuego that reports 30.5 ind/km^2^ [60], a value one order of magnitude larger than ours. However, this study is based on surveys performed from roads, a strategy known to violate several assumptions of design based estimations [61].

The reported relationship between guanaco abundance and environmental factors would mainly reflect the response of guanaco to climatic seasonality. During the non-breeding season, abundance increases at lower elevation areas (Figs 2 and 4A) meanwhile, during the breeding season, abundance increases towards the southwest, which is reflected in the response to the geographic location (Fig 3B), even where the elevation reaches its highest values [39]. This relation supports the hypothesis of seasonal displacements of the guanaco in Tierra del Fuego, with guanacos moving to lower elevation areas outside the breeding season and returning to higher elevation areas during the breeding season [62]. Seasonal displacements in response to elevation have been observed in other wild ungulates inhabiting mountain areas such as the mule deer (*Odocoileus hemionus hemionus*) [21], the red deer (*Cervus elaphus L*) [29, 30] and the roe deer (*Capreolus capreolus*) [63].

Some authors suggested that high snow cover and snow storms trigger guanaco movements to lower areas during the non-breeding season, maybe due to the reduction of food availability [37, 40]. However, the triggering mechanism for displacement has not been clearly identified. Three mechanisms could contribute, alone or together, to drive movements. First, harnessing forage at an early ripening stage would be advantageous for the guanaco, as has been proposed for other mountain herbivores [29, 64, 65]. Second, the use of certain sites in territorial herbivores is controlled by the dominant social class, forcing the movement of subordinate individuals to other areas by the so-called "social fences" [66]. The guanaco presents a strong social structure formed by family groups, where the male defends a territory from other males during the breeding season [36]. Then, during the breeding season, the male of the family groups accentuates their territorial behaviour [36, 67] and would force the dispersion of subordinate individuals to new areas [66].Third, a denso-dependence mechanism as competition for resources could also be forcing displacement of individuals from wintering areas towards elevated areas [63].

In spite of the seasonal displacement revealed by our analysis, the guanaco distribution reveals zones of high abundance throughout the year (Northeast and Southeast, Fig 4A and 4B) and zones with high and low abundance in opposite seasons (Southwest, Fig 4A and 4B). This could be a reflection of partial migrations, as it was already described by Raedeke for the guanaco in Chilean Tierra del Fuego [36]. Thereby, we propose that guanacos from the Southwest of our study area move seasonally in response to elevation meanwhile those located in the Southeast and Northeast are more sedentary. Then, the higher zones at the Southwest would be part of the guanaco habitat range only during summer.

The general distribution pattern of guanaco described here is consistent with previous information for the Argentine portion of the Isla Grande Tierra del Fuego. An example is the fact that the large river’s valleys (La Turba, Ona, Candelaria, Ewan, San Pablo) (Fig 1) presented the highest densities during the non-breeding season (Fig 4A). The San Pablo River valley is historically recognized as one of the “corridors” for guanacos moving from the mountain range to low areas during the winter months [44]. In the same way, the Central-East zones of the “Ecotono” were perceived by local people as important wintering areas [53]. However, livestock ranching, the main activity in the study area [68] could also explain the distribution of guanaco, mainly during winter, when the low zones present larger densities.

The selected models explained around 25% of the guanaco abundance variability (26.2% in breeding season and 24.2% in non-breeding season). However, the use of GAMM as a final model for the non-breeding season didn't allow us to assess the explained deviance. For this season, we assume that the fit of the model using GAMM was similar to its GAM based on the variation coefficient, which was low and similar for the two models (15% and 12% respectively). In turn, the explained deviance of the selected models (Table 2) was similar to that obtained for the guanaco in *Reserva La Payunia* [58]. Based on the low variation coefficients reported here, our estimates can be considered accurate. The assumption that most of the guanaco's groups are found in the open pasture areas during daytime could underestimate the abundance, as animals located in the forests wouldn’t be detected. However, it is known that guanacos perform daily movements in the studied area [36]. Guanacos sleep and look for refuge in the forest by night and move to the open areas during the day, where they can feed and be vigilant. Future studies must assess the validity of this assumption and will help to adjust assessments made form open areas [69]. We also highlight that the use of Density Surface Modelling has advantages over design-based methods, where samples need to be allocated in the survey area ensuring an even coverage probability to allow extrapolation to the unsurveyed area [14]. In addition, Density Surface Modelling uses spatially explicit modelling to incorporate the effect of environmental heterogeneity in estimation and scaling up from the covered region. Indeed, it’s possible to infer abundance using non-randomized surveys or opportunity platforms. A good example of this is the Williams' study [70], that used line transect data coming from touristic cruises and applied GAMs to describe count data of cetaceans as smooth of spatial environmental covariates, for predicting density throughout the study area.

According to our study, the valleys would be important landscape features during the winter, where guanacos would move to feed themselves and, perhaps, use them as corridors to access the less severe areas near the coast. If part of the population of guanacos carries out seasonal migrations, it would be important to know the range they cover, the migration routes and if there are stopovers. When the survival and reproductive success of migratory species depends on the quality of spatially different habitats, it is essential to incorporate this knowledge in conservation actions [5] to achieve conservation targets avoiding problems of scale mismatch, i.e. when the scales of the intervention actions and of the ecological processes or natural resources being managed are not aligned [71]. For example, the zonation established for keeping a low impact by elephants in the Kruger National Park failed because they did not consider the elephant’s spatial and temporal use of the area [71]. In an analogue way, a conservation target of maintaining the habitats required for guanacos would demand including the areas involved in the seasonal movement of animals.

The study of species distribution patterns has been applied for purposes as diverse as to determine areas of high density of individuals [70] or of high species diversity [15], to optimize disease vector control operations [72], to control the expansion of invasive species [73] or to detect suitable habitat conditions [50, 74]. Our study of the distribution of abundance of guanaco allows to detect habitats of seasonal importance, which could be a useful example to apply to other species of large herbivores that carry out migratory movements in response to environmental heterogeneity.

## Supporting information

S1 Dataset Count data. (TXT)

S2 Dataset Segment location. (TXT)

S1 Photographs Surveys examples of guanaco’s groups at large distances (Ph1, 885 m) and small distances (Ph2 and Ph3, 257.30 and 275.35 m) to the flight path. Survey of solitary individuals at a great distance (Ph4, 558.37 m) and at a small distance (Ph5, 117.75 m) to the flight path. Helicopter photo (Ph6). View from inside the helicopter (Ph7-9, right side of the helicopter; Ph10-11, left side of the helicopter).
(RAR)

S1 File The procedure followed to assess the bandwidth. (DOCX)
