# Supplementary material for: Spatial abundance models and seasonal distribution for guanaco (Lama guanicoe) in central Tierra del Fuego, Argentina
Source: PLoS One. 2018 May 21;13(5):e0197814. doi: 10.1371/journal.pone.0197814 (PMC5962061; doi:10.1371/journal.pone.0197814)
Supplement: S1 File — (DOCX) [file pone.0197814.s004.docx]

**The procedure followed to survey and to assess the bandwidth**

Strip transects are a special case of line transects surveys, when all objects inside a strip of a defined width can be counted. “Large mammals that are readily seen from the air can be censused most effectively from aerial surveys that utilise light aircraft or helicopters. The most common method involves flying strip transects and counting all the individuals within the strip” (Sutherland 2006). This is the case of the guanacos, large herbivores of about 90 kg in weight and 120m in height at the shoulder, easily distinguished from the air. Using strip transects, it is understood that all the animals inside the defined strip are visible.

As Buckland *et al.* (2015) pointed out, “helicopters offer some advantages for aerial surveys because they give less obstructed views forward and below the aircraft, thereby reducing the need for an offset, as well as reducing the difficulties of responsive movement if the observer is able to detect animals before they respond to the aircraft”. Also, helicopters flight at low speed and altitude than conventional aircrafts, reducing the missing of objects derived from the speed at heights that airplanes are forced to fly.

Then, for large herbivores like the guanaco, surveying them by helicopter represents an opportunity to improve estimations. The key point then is how to define the width of the strip to be applied in surveys and analysis.

Band width was set at 700 m after we performed an analysis on a set of pictures, to assess how the detection process might affect our estimations. As pictures are taken from the helicopter to the sides, the question is how far away from the helicopter the pictures allow to identify and count guanaco groups, to use strip transect as an analysing strategy.

A set of 40 pictures of guanaco groups taken from the helicopter during the survey of 14 February 2008, were located geographically in the landscape using Google Earth. We selected a group of pictures taken in a part of the survey area where landscape features allow locating a picture on the terrain. The presence of conspicuous and identifiable features like forest patches boundaries, watercourse meanders, peat bogs and bush patches allowed to locate the position of guanaco groups in the landscape. The process is tedious but allows locating precisely a picture in a geographic location in this landscape.

Guanaco groups positions were transferred to a GIS. The distance from the group to the flight line was assessed using the function NEAR in ArcGis 10.3.1 (ESRI). These distances assimilate to the perpendicular distances from the survey line to the object of interest, used in the distance based estimation methods. The farthest picture was taken at 885m from the flight line.

With these distances we assessed the detection function in the software DISTANCE. We compared four different models traditionally used in analysis for distance data. Uniform without adjustment term (strip transect), Uniform with cosine adjustment, Heminormal with simple polynomial and Hazard rate with Hermite polynomial (Table 1). Distance data were transformed in 100 m intervals for analysis and to allow comparison between models. Right truncation was set at 700 m (six observations were discarded) as it is known that truncation increases robustness of the fit for the models, and that sightings far away from the line contribute little to fit of the model at small distances, which is where fit is important (Buckland *et al*. 2015).

Usually the best model is selected comparing the values of the Akaike Information Criterion (AIC), and choosing the model of lowest AIC value (Burnham and Anderson 2003). If the difference between AIC for compared models is less than 2, then there is no evidence in favour of selecting one model against other. In our case, as the difference in AIC is less than 2, so there is no evidence to choose one model against other. Models with similar AICs will provide similar estimates probabilities of detection. Then, there is little difference in selecting between these models.
Following the comparison between models, the Uniform model implies (obviously) no parameter, against the Uniform + cosine and Heminormal + simple polynomial, who present, each one, one parameter in the model, and the Hazard Rate + Hermite polynomial, who presents two parameters in the model (Table 1). As Burnham and Anderson (2002, page 31, following Box and Jenkins) point out, “the principle of parsimony should lead to a model with “. . . the smallest possible number of parameters for adequate representation of the data.” Statisticians view the principle of parsimony as a bias versus variance tradeoff “.

Another way to compare between model is the Goodness of Fit test. The four models reveal to adjust similarly when comparing p values for statistics (Table 1) and the graphs of the detection functions fitted to data (Figures 1 to 4). However, is must be taken into account that this test is incapable of discriminating between quite different models near the line, the most critical region (Buckland et al. 1993).

Taking into account the difference in AIC values and the number of parameters for each model, choosing a Uniform function will provide a reasonable estimation of the detection function. Then, we assume that a bandwidth of 700 m is a reasonable width for surveying large mammals like the guanaco in the context of the field work we carried out.

**Table 1. Models used to assess the detection function and its evaluation parameters. Ordered by increasing difference in AIC.**

| **Model** | **Delta AIC** | **AIC** | **Number of parameters** | **Effective Strip Width** | **p of Goodness of fit test** |
| --- | --- | --- | --- | --- | --- |
| Uniform + cosine | 0 | 131.36 | 1 | 493 | 0.675 |
| Heminormal + Simple polyinomial | 0.25 | 131.61 | 1 | 518 | 0.643 |
| Uniform | 0.96 | 132.32 | 0 | 700 | 0.478 |
| Hazard + Hermite polynomial | 2.23 | 133.59 | 2 | 499 | 0.517 |


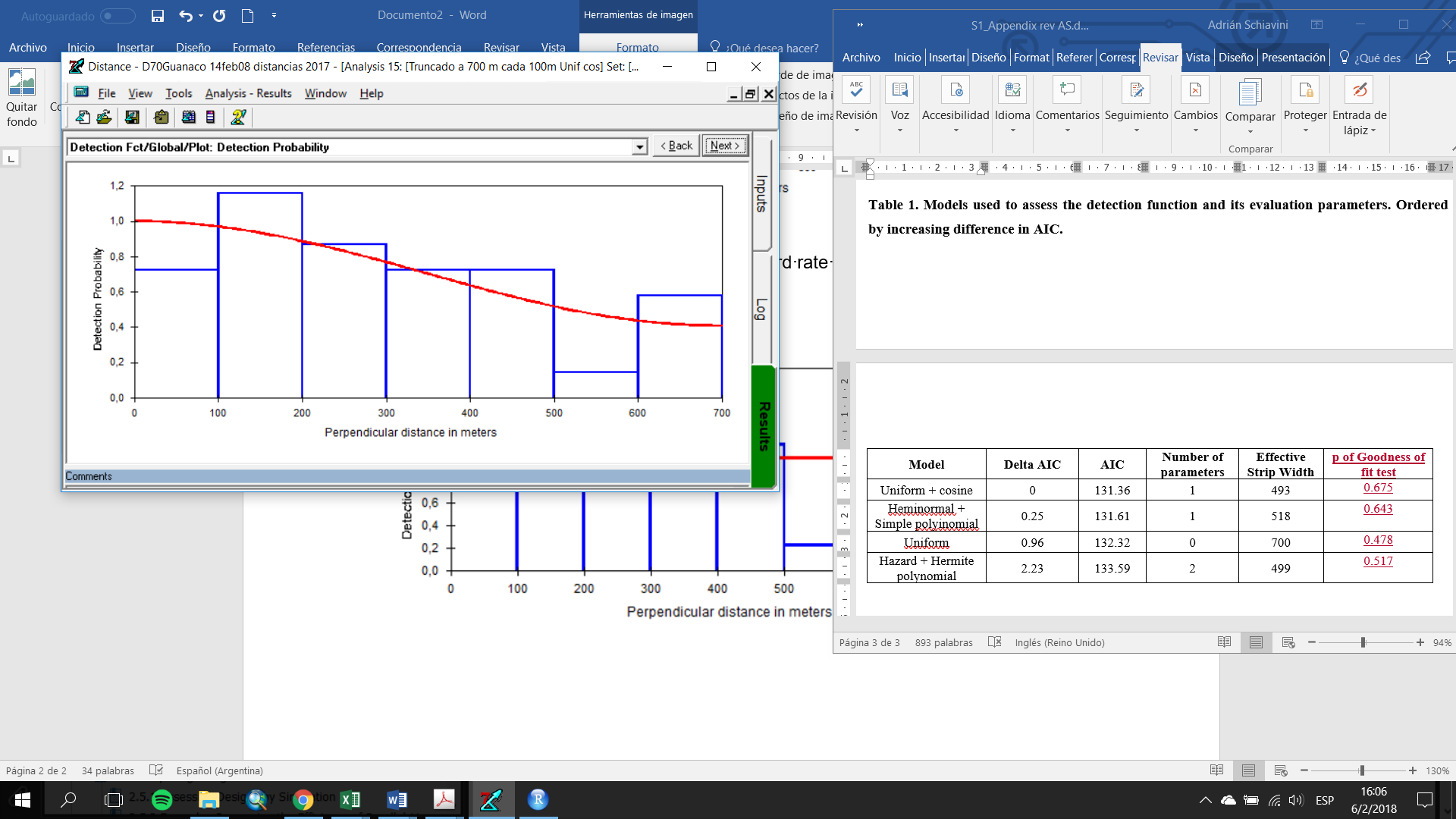


**Figure 1.** Detection probability plot. Adjusted function Uniform with cosine adjustment terms.


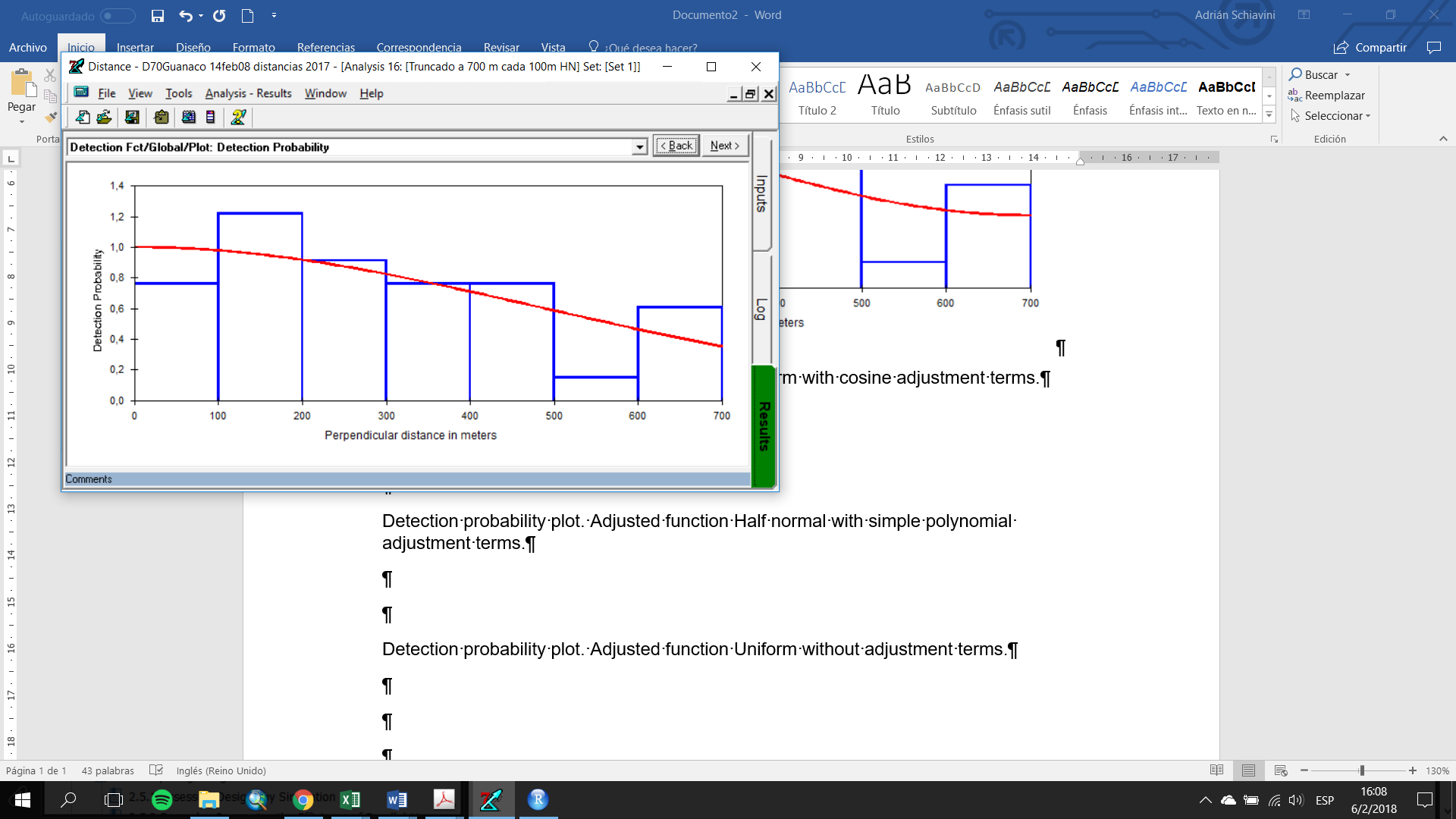


**Figure 2.** Detection probability plot. Adjusted function Half normal with simple polynomial adjustment terms.


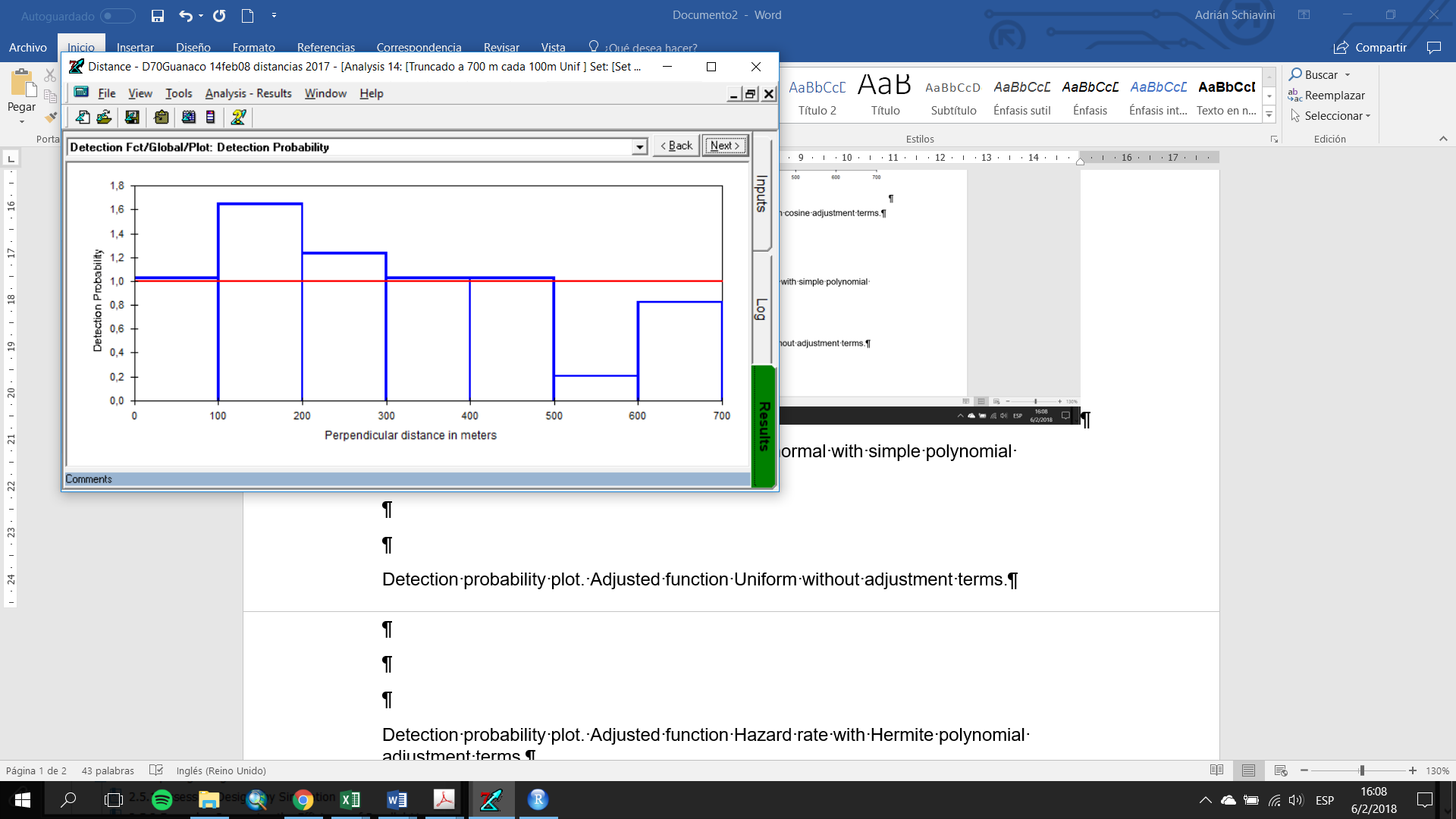


**Figure 3.** Detection probability plot. Adjusted function Uniform without adjustment terms.


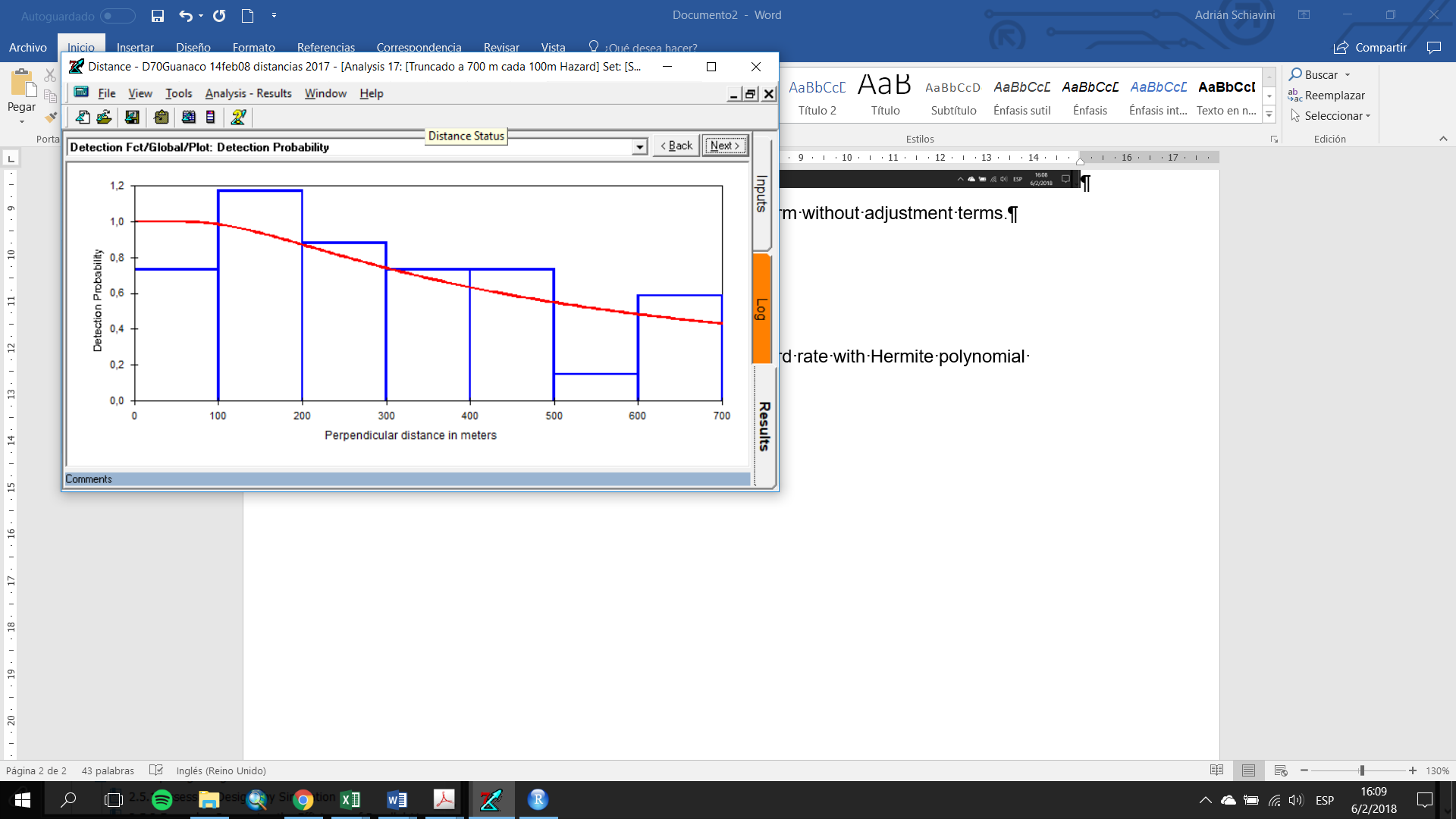


**Figure 4.** Detection probability plot. Adjusted function Hazard rate with Hermite polynomial adjustment terms.
